# Steering Bioelectrochemical CO_2_ Reduction Toward Methanogens Suppression and Acetogen Bioaugmentation

**DOI:** 10.3390/bioengineering13091023

**Published:** 2026-09-02

**Authors:** Jacopo Ferretti, Angela Marchetti, Marco Zeppilli

**Affiliations:** Department of Chemistry, Sapienza University of Rome, Piazzale Aldo Moro 5, 00185 Rome, Italymarco.zeppilli@uniroma1.it (M.Z.)

**Keywords:** *Acetobacterium woodii* bioaugmentation, bioelectrochemical systems, CO_2_ bioreduction, microbial electrosynthesis, organic acids production, methanogenesis suppression

## Abstract

Biological strategies for converting carbon dioxide (CO_2_) into valuable compounds are attractive approaches for a carbon-neutral future. Bioelectrochemical systems (BESs) represent an innovative strategy for the control of microbial metabolism, in which electrochemical techniques are adopted to stimulate reductive and oxidative processes. Acetogenesis and methanogenesis are the two main chemoautotrophic pathways of CO_2_ reduction usually present in anaerobic environments. Due to the syntrophic and competitive relationship between acetogens and methanogens, methanogenesis inhibition strategies should be adopted to direct CO_2_ reduction towards acetate and fatty acids. In this work, an acetogen-enriched inoculum was produced by the bioaugmentation of *Acetobacterium woodii* in the heat-shocked and acid-treated inoculum. Then, by using H-cell reactors, without the use of any chemical inhibitor, this inoculum was tested in semi-continuous mode by imposing a dilution rate previously identified from growth kinetic assessment. Bioelectrochemical tests, conducted at −0.9 V and −0.7 V vs. SHE, showed the overcoming of acetogenesis on methanogenesis. At −0.7 V vs. SHE, acetate was produced at 0.0385 ± 0.009 mmol d^−1^ and 0.0343 ± 0.010 mmol d^−1^ in the absence and presence of bioaugmentation, respectively, with acetate cathodic coulombic efficiency (CCEs) of 68% and 64%. At −0.9 V vs. SHE, bioaugmentation markedly reduced methanogenesis, decreasing the methane production rate from 0.105 ± 0.012 to 0.007 ± 0.004 mmol d^−1^, while acetate production reached 0.061 ± 0.025 mmol d^−1^ with a CCE of 43%. Finally, the effect of bioaugmentation was demonstrated by cyclic voltammetry of the biocathode, which showed the increase in biocatalytic activity due to the presence of *Acetobacterium woodii*.

## 1. Introduction

Anthropogenic carbon dioxide (CO_2_) emissions remain one of the major contributors to global climate change, highlighting the urgent need for sustainable technologies capable of mitigating atmospheric CO_2_ accumulation while promoting resource recovery [1]. Rather than considering CO_2_ solely as a waste product to be captured and stored, increasing attention is being devoted to its biological conversion into value-added chemicals within the framework of carbon capture and utilization (CCU) [1,2]. This approach is fully aligned with the transition towards a circular bioeconomy, in which carbon-containing waste streams are transformed into renewable resources through environmentally sustainable processes. Compared with conventional thermochemical routes, biological CO_2_ conversion operates under mild conditions and requires significantly lower energy inputs, making it an attractive alternative to produce bio-based chemicals [2]. Acetogenesis and methanogenesis are the two predominant autotrophic pathways involved in this microbial CO_2_ reduction carried out by anaerobic mixed microbial cultures (MMCs). Both pathways rely on the Wood–Ljungdahl pathway for carbon fixation but lead to markedly different products and process outcomes [3]. While methanogenesis converts CO_2_ into methane, acetogenesis produces acetate, one of the most important platform chemicals in the emerging bioeconomy. Indeed, besides its direct industrial applications, acetate can serve as a building block to produce biopolymers, or even, for the microbial production of medium- and long-chain carboxylic acids through chain elongation, substantially increasing the value of recovered carbon [4,5,6]. Nevertheless, the recovery of fatty acids from waste streams is gaining increasing interest as a strategy for valorizing residual organic matter and generating valuable intermediates for subsequent biotechnological processes [7,8].

Consequently, promoting acetogenesis over methanogenesis represents a key objective for maximizing the efficiency and economic viability of biological CO_2_ valorisation. However, selectively directing MMCs towards acetogenesis remains a major challenge, due to the methanogens thermodynamic advantage over acetogens, thus dominating the conversion of CO_2_ under favourable operating conditions [9,10]. Several approaches have been proposed to overcome this limitation, including the addition of methanogenic inhibitors, biomass pretreatments and the use of pure acetogenic cultures [11,12,13]. Chemical inhibitors such as 2-bromoethanesulfonate (BES/BESA) effectively suppress methane production but increase operating costs and may interfere with the microbial community, limiting their applicability in large-scale processes [14,15]. Alternatively, thermal and acid pretreatments exploit the higher resistance of spore-forming acetogens to selectively reduce methanogenic populations [16]. Although these approaches successfully delay methane production, previous studies have shown that methanogenesis often recovers over time, particularly under continuous or semi-continuous operation [16,17]. Pure acetogenic cultures overcome competition with methanogens but require sterile operating conditions and strict process control, significantly increasing operational complexity and limiting practical scalability [18,19]. These limitations highlight the need for alternative strategies capable of maintaining stable acetogenic activity in mixed cultures without relying on chemical inhibition. In this regard, microbial enrichment and bioaugmentation represent promising approaches to reinforce acetogenic populations while preserving the robustness and operational simplicity of mixed microbial communities [20]. Among acetogenic microorganisms, *Acetobacterium woodii* has been extensively investigated as a model homoacetogen because of its well-characterized ability to grow autotrophically using H_2_ and CO_2_ and to convert these substrates predominantly into acetate through the Wood–Ljungdahl pathway [21]. This metabolic capability makes *A. woodii* particularly attractive for CO_2_ valorisation strategies aimed at directing carbon and reducing equivalents towards acetate production. Moreover, its well-characterized physiology and established use in CO_2_/H_2_-based bioprocesses make it a suitable organism for bioaugmentation of mixed microbial communities [22]. In the context of bioelectrochemical systems, *A. woodii* has also been investigated as a component of microbial electrosynthesis (MES) communities, although direct electron uptake from the electrode has not been demonstrated for this species [23]. More recently, an *A. woodii*-enriched MES system achieved acetate production of 17.4 g L^−1^ with a coulombic efficiency of 62%, further highlighting the potential of this acetogen for bioelectrochemical CO_2_ valorisation [24]. Therefore, in the present study, *A. woodii* was selected as a bioaugmentation strain to increase the acetogenic fraction of the mixed inoculum and potentially enhance the competition of acetogenesis with methanogenesis under bioelectrochemical conditions.

Among the available biological technologies, bioelectrochemical systems (BESs) have emerged as a promising platform for microbial CO_2_ valorisation [25,26]. By exploiting the ability of electroactive microorganisms to exchange electrons with solid electrodes through extracellular electron transfer (EET), BES enable the electrochemical control of microbial metabolism, allowing the selective reduction in organic and inorganic carbon source into valuable products [27,28]. Unlike conventional hydrogen-mediated gas fermentation, where externally supplied hydrogen suffers from low solubility and handling limitations, BES generate reducing equivalents directly at the cathode surface, enhancing electron availability for autotrophic microorganisms and improving process efficiency [29,30]. Nevertheless, the long-term stability of enriched MMCs and their ability to maintain selective acetogenesis under bioelectrochemical conditions remain insufficiently understood, particularly when methanogenesis is controlled exclusively through operational parameters rather than chemical inhibitors. In this context, the present study investigated the combined effect of biomass pretreatment, bioaugmentation with *Acetobacterium woodii*, and bioelectrochemical process control on the selective conversion of CO_2_ into carboxylic acids using MMCs. Attention was devoted to evaluating whether acetogenesis could be maintained under semi-continuous hydrogenophilic and bioelectrochemical conditions without the addition of chemical methanogenic inhibitors. By integrating microbial enrichment with the optimization of key biolectrochemical operational parameters, this work provides further insight into the development of sustainable strategies for steering CO_2_ conversion towards valuable products while preserving the advantages associated with mixed-culture systems.

## 2. Materials and Methods

### 2.1. Inoculum Preparation

To obtain a microbial consortium with high metabolic diversity and maximize the potential for acetogenic enrichment, a mixture of activated sludge and anaerobic digestate, collected from a real scale wastewater treatment plant located in the northern-east Italy, was used as the starting inoculum, with a ratio of 1:1. The sludge mixture was first dried in the oven at 70 °C until complete dehydration and subsequently subjected to thermal pretreatment at 120 °C for 2 h in a muffle furnace following the method reported elsewhere [31,32]. The heat-treated biomass was then suspended into an acid solution at pH 2 for 48 h, thus obtaining a thermal/acid-pretreated inoculum. These pretreatments were applied to suppress methanogenic activity while selecting spore-forming microorganisms, including acetogenic bacteria, which can withstand severe thermal and acidic conditions [33,34]. Finally, two 200 mL inocula were obtained, one of which bioaugmented with 10 mL of active culture *Acetobacterium woodii* DSM 1030 (DSMZ, Braunschweig, Germany), thus obtaining acetogen-enriched mixed culture. Prior to start with the H-cell tests, all inocula were activated under mixotrophic conditions for 10 days using a gas mixture supplemented with 2 g L^−1^ glucose and 2 g L^−1^ peptone (Sigma-Aldrich, St. Louis, MO, USA). In detail, the gas mixture used for inoculum selection was prepared from pure H_2_, CO_2_, and N_2_ gases and stored in Tedlar^®^ gas sampling bags (Supelco, Merck/Sigma-Aldrich, Darmstadt, Germany). The resulting mixture consisted of 50% H_2_, 35% N_2_, and 15% CO_2_ (*v*/*v*). Before each incubation period, the headspace of the serum bottles was flushed with the prepared gas mixture to provide the required H_2_ and CO_2_ sources and to establish the desired anaerobic conditions.

### 2.2. Operative Conditions of H-Cell Tests

Following the inoculum preparation and incubation period, bioelectrochemical experiments were carried out in laboratory-scale H-cell reactors, consisting of two 250 mL (working volume 200 mL) borosilicate glass chambers connected through a side flange and separated by a 0.01 m^2^ anion exchange membrane (AEM; FUMASEP^®^ FAS, Fumatech GmbH, Bietigheim-Bissingen, Germany). These systems worked for 40 days under autotrophic conditions using the mineral medium without the addition of organic carbon sources, with CO_2_ serving as the sole carbon source and H_2_ as the electron donor for autotrophic growth. The mineral medium was composed of: NH_4_Cl (0.125 g/L); MgCl_2_ · 6H_2_O (0.1 g/L); K_2_HPO_4_ (4.0 g/L); CaCl_2_ · 2H_2_O (0.05 g/L); 10 mL/L of a trace metal solution and 1 mL/L of vitamin solution [35].

**Figure 1:**
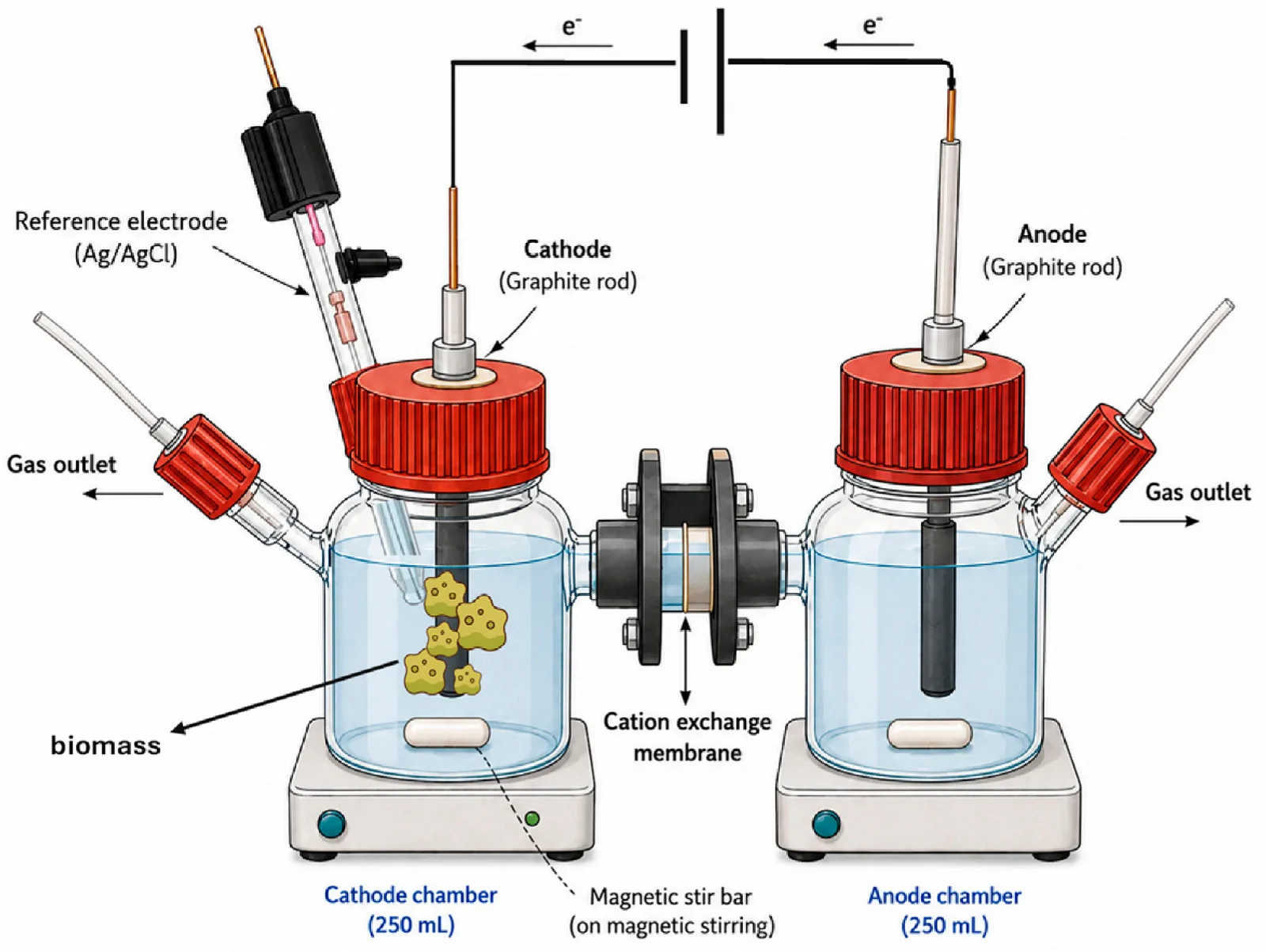
Schematic illustration of the H-cell reactors used during the semi-continuous tests.

The reactors were operated in a three-electrode configuration, with the cathode serving as the working electrode (WE), the anode as the counter electrode and an Ag/AgCl reference electrode (Amel S.r.l., Milan, Italy), immersed in a saturated KCl solution with E°’ = 199 mV vs. the standard hydrogen electrode (SHE), positioned close to the cathode. Both the anode and cathode were graphite rod electrodes (electrode surface 11 cm^2^), with a cathode potentials controlled at −0.7 and −0.9 V vs. SHE, by using a potentiostat (BioLogic Science Instruments, Seyssinet-Pariset, France). A schematic representation is reported in Figure 1. Cyclic voltammetries were conducted using a scan rate of 0.083 mV/s varying the potential from −0.5 to −1.5 V vs. Ag/AgCl for a total of 3 cycles.

Both chambers were filled with the same mineral medium to maintain electroneutrality across the membrane. The anode chamber was operated under abiotic conditions, whereas the cathode chamber was inoculated with biomass obtained from the enriched cultures. Specifically, 100 mL of inoculum, with a concentration of 50 mgVS/L, was centrifuged (8000 rpm, 15 min), and the resulting biomass was resuspended in fresh mineral medium containing no organic substrates before being transferred to the cathodic compartment. Prior to semi-continuous operation, the bioelectrochemical reactors were operated in batch mode for seven days to determine the maximum specific growth rate (μ_max_) under bioelectrochemical conditions.

All experiments were conducted at room temperature, approximately 25 °C, under continuous magnetic stirring. The reactors were operated in semi-continuous mode by replacing 43 mL per day of culture medium with fresh mineral medium; the corresponding HRT resulted 4.6 days. This operating condition was selected to maintain a dilution rate (D_rate_) corresponding to 50% of the experimentally μ_max_ that was determined during the first seven days of reactor operation and subsequently used to define the daily D_rate_. Daily medium replacement was adopted to favour the washout of methanogenic microorganisms while minimizing their attachment to the cathode surface. After each feeding cycle, the reactor headspace was flushed with a N_2_/CO_2_ gas mixture (30:70, *v*/*v*), thus ensuring the anaerobic conditions. At the end of the experimental period, the electrochemical activity of the biocathodes was evaluated by a potenziodynamic technique, i.e., cyclic voltammetry, using scan rates of 5, 10, 20 and 40 mV s^−1^ over a potential window ranging from −0.5 to −1.5 V vs. SHE.

### 2.3. Analytical Procedures

The composition of the gaseous phase (H_2_, CO_2_, and CH_4_) was determined by gas chromatography (DANI MASTER, Dani Instruments S.p.A., Milan, Italy). Gas samples (50 μL) were collected from the reactor headspace using a gas-tight Hamilton syringe (Hamilton Company, Reno, NV, USA) and injected into a stainless-steel column packed with a molecular sieve. Nitrogen was used as the carrier gas at a flow rate of 18 mL min^−1^. The oven temperature was maintained at 180 °C, while the thermal conductivity detector (TCD) was operated at 200 °C [36]. H_2_, CO_2_, CH_4_ calibration curve was performed using calibration curves between 0 and 60% of each analyte. The concentration of carboxylic acids in the liquid phase was determined using a gas chromatograph (Agilent 8860 GC, Agilent Technologies, Santa Clara, CA, USA) equipped with a flame ionization detector (FID). Prior to analysis, liquid samples were filtered through 0.20 μm cellulose acetate syringe filters (25 mm diameter) (Whatman, Maidstone, UK) and acidified with 37% (*w*/*v*) phosphoric acid. A sample volume of 1 μL was injected into the GC system. The oven temperature program was as follows: 60 °C for 1 min, followed by a temperature ramp of 20 °C min^−1^ up to 150 °C, and finally held at 175 °C for 1.25 min. N_2_ was used as the carrier gas, while the injector and flame ionization detector (FID) (Dani Instruments S.p.A., Milan, Italy), temperatures were maintained at 200 °C and 320 °C, respectively. Acetate, propionate, butyrate, iso-butyrate and valeric acid calibration curve was realized using a multi-standard solution in the range of 0–200 mg/L of each analyte. Biomass growth was monitored spectrophotometrically by measuring the optical density at 600 nm (OD_600_). Optical density values were converted into volatile suspended solids (VSS) concentrations using specific calibration curves (10–200 mgVS/L), according to standard methods [37]. Biomass concentrations were determined experimentally as VSS and subsequently converted into molar units using an assumed biomass molecular weight of 113 g mol^−1^ [38].

### 2.4. Experimental Parameters and Calculation Methods

Under semi-continuous H-cell operation, the daily replacement of the liquid phase allowed the system to reach pseudo-steady-state conditions. Therefore, biomass growth and carboxylic acid production rates were calculated by applying the mass balance of a continuous stirred reactor, where Q represents the daily liquid flow rate (L d^−1^):

(1) $$r_{\mathrm{Acids};\mathrm{Biomass}}\left(\frac{\mathrm{mmol}}{d}\right)=\left[Acid;\;Biomass\right]*Q$$

The H-cell reactors were initially operated in batch mode for seven days to determine the μ_max_ of the bioelectrochemical cultures. The obtained value was subsequently used to define the D rate applied during semi-continuous operation.

Under steady-state conditions, the D rate of a continuous reactor was related to the specific growth rate according to:

(2) $$\;rate\;\left(d^{-1}\right)=\frac{Q}{V}=\;µ$$

where V is the reactor working volume (L). The critical dilution rate corresponds to the maximum specific growth rate is:

(3) $$D_{crit}\left(d^{-1}\right)=\;{µ}_{max}$$

For the Bioelectrochemical tests, μ_max_ was estimated from the exponential increase in biomass concentration observed during the initial 7-day batch phase according to:

(4) $$µ\;\left(d^{-1}\right)=\frac{1}{X}\frac{dX}{dt}$$

where X is the biomass concentration expressed as gCOD L^−1^, converted into COD equivalents using a conversion factor of 1.42 gCOD gVSS^−1^, based on the empirical biomass formula C_5_H_7_O_2_N [39]. The value obtained from Equation (4) was used as the μ_max_ representative of the bioelectrochemical cultures and served as the basis for setting the appropriate operating D rate, maintaining microbial growth under exponential conditions during the semi-continuous experiments.

The coulombic cathodic efficiency (CCE) was calculated according to Equation (5) and represents the fraction of electrons (e^−^), measured through the external circuit of potentiostat, that was recovered during the working days in the reduction products derived from CO_2_ conversion, namely methane and carboxylic acids:

(5) $$CCE\;\left(\%\right)=\frac{\mathrm{meq}\;\left({\mathrm{CH}}_{4};\;{\mathrm{CH}}_{3}\mathrm{COOH}\right)}{\int_{t=n}^{t=n+1}e^{-}dt}*100$$

In particular, the electrical charge transferred through the external circuit was converted into a milliequivalent amount using Faraday’s law and subsequently expressed as COD.

Finally, the efficiency ratio (ER), calculated according to Equation (6), was used as an indicator to evaluate the effect of bioaugmentation on carboxylic acid production and on the selective promotion of acetogenesis over methanogenesis:

(6) $$ER\;\left(\%\right)=\frac{{\%}_{Total\;acids;\;\;Methane\;Bioaugmentation}}{{\%}_{Total\;acids;\;\;Methane\;No\;bioauemgntation}}$$

## 3. Results

### 3.1. Influence of Cathodic Potential and Bioaugmentation on CO_2_ Reduction in Bioelectrochemical Reactors

The performance of the bioelectrochemical reactors was evaluated under semi-continuous operation by comparing two cathodic potentials, −0.7 and −0.9 V vs. SHE, using inocula with and without *Acetobacterium woodii* bioaugmentation. All reactors were operated in batch mode for seven days to determine the μ_max_, which was subsequently used to establish the D rate for semi-continuous operation. Similar μ_max_ values were obtained under both cathodic potentials (0.413 ± 0.016 d^−1^), indicating comparable initial biomass growth kinetics regardless of the applied potential.

**Figure 2:**
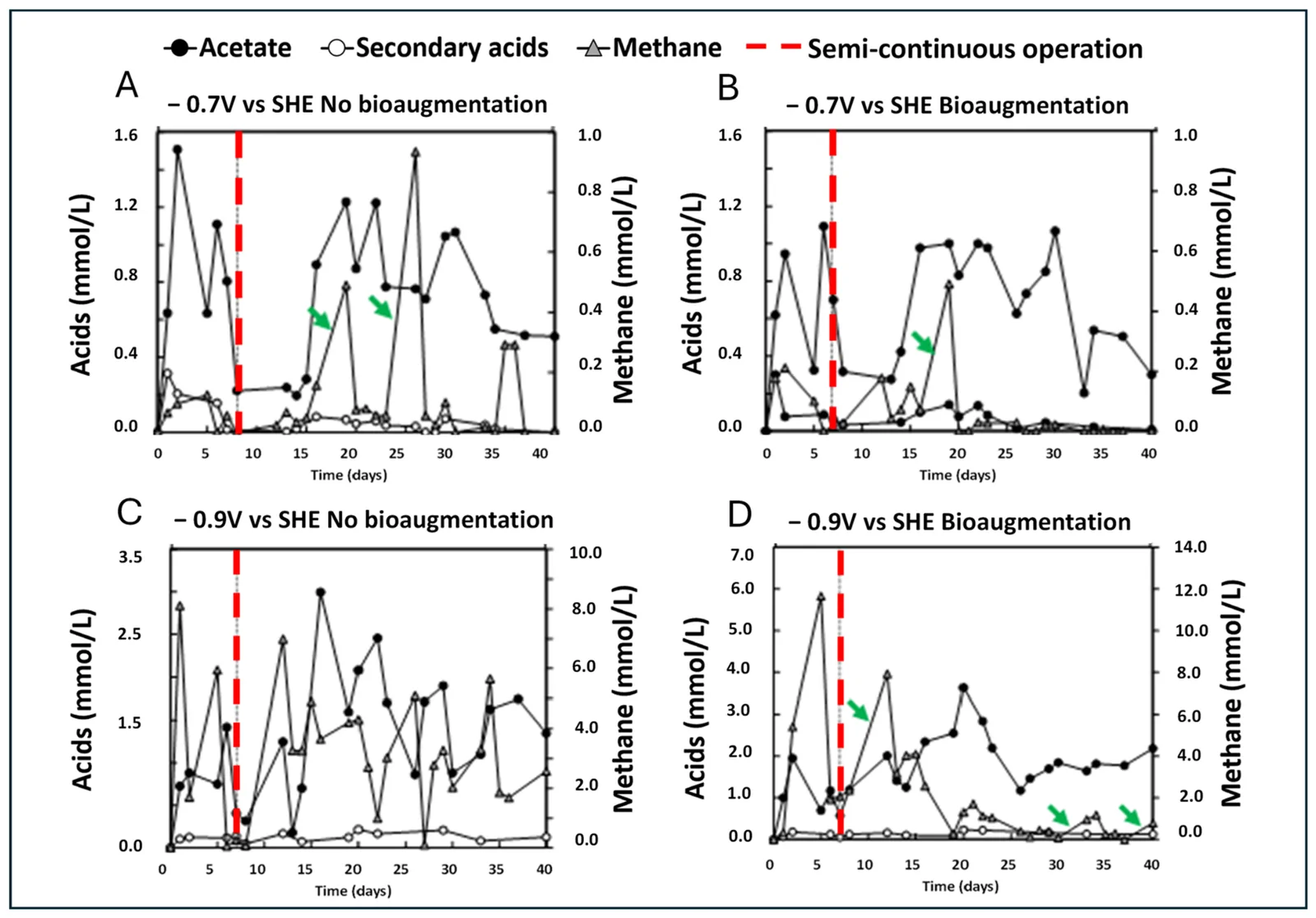
Temporal evolution of acids and methane concentration during semi-continuous operation of the H-cell bioelectrochemical reactors under different cathodic potentials and inoculum configurations. Panels show the concentration profiles obtained at −0.7 V vs. SHE (**A**,**B**) and −0.9 V vs. SHE (**C**,**D**) using non-bioaugmented (No Bioaugmentation) and bioaugmented (Bioaugmentation) mixed-culture inocula containing *Acetobacterium woodii*. The dashed vertical line indicates the transition from the initial batch phase to semi-continuous operation. The green arrows indicate weekends during which daily medium replacement and headspace renewal were not performed.

The temporal evolution of acetate, secondary carboxylic acids, and methane concentrations under the four investigated operating conditions is shown in Figure 2A–D. The transition from the initial batch phase to semi-continuous operation resulted in markedly different concentration profiles depending on the applied cathodic potential and the presence of *A. woodii* bioaugmentation. Table 1 reports the average current density and cell voltage utilized in the different H cells.

At −0.7 V vs. SHE, both reactors exhibited acetate production throughout the experimental period, equal to 0.85 ± 0.1 mmol L^−1^, whereas methane concentrations after the transition to semi-continuous operation, remained negligible, with production rates of 0.0039 ± 0.001 mmol d^−1^ in the non-bioaugmented H-cell reactors and 0.0002 ± 0.001 mmol d^−1^ following bioaugmentation. Secondary carboxylic acids were detected only at low concentrations under both bioaugmented and non-bioaugmented conditions, with a predominance of valeric acid, indicating that acetate remained the dominant product of CO_2_ reduction at this potential.

**Figure 3:**
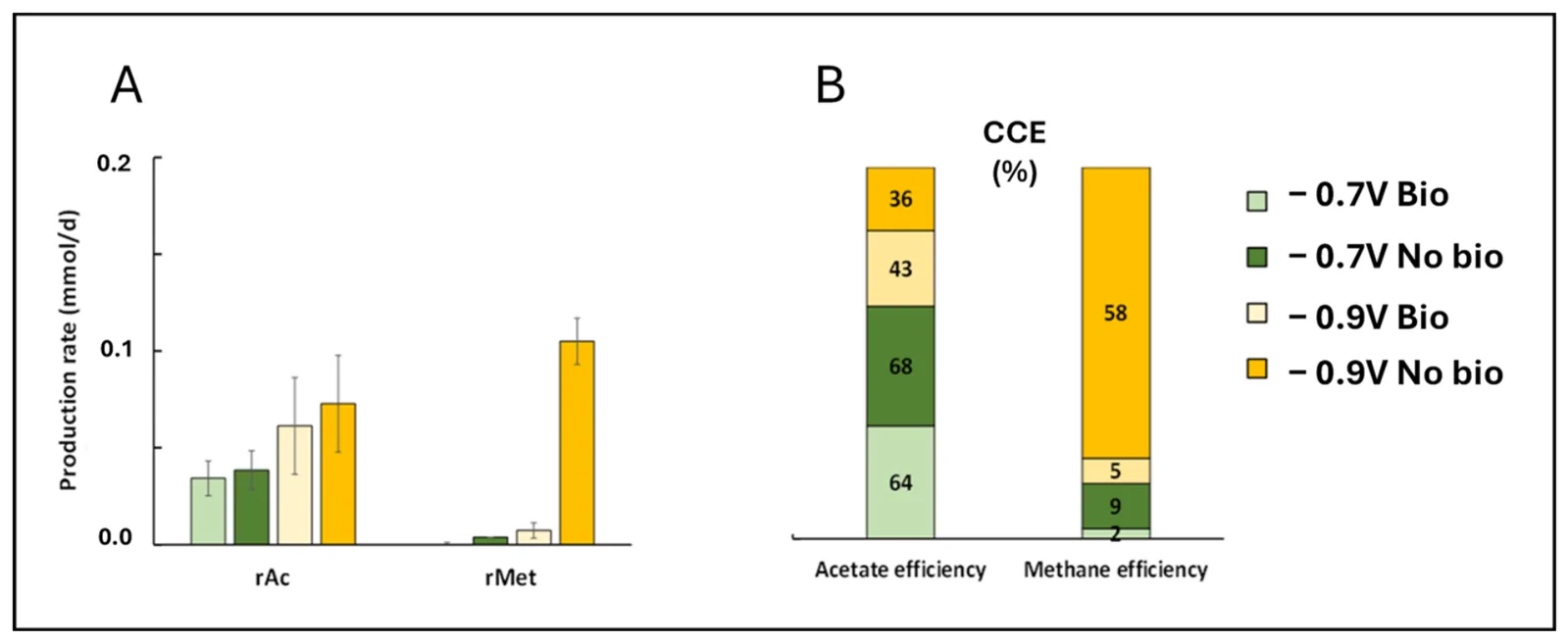
Quantitative comparison of reactor performance under different cathodic potentials and inoculum configurations. (**A**) Average production rates of acetate and methane during semi-continuous operation of the H-cell bioelectrochemical reactors. (**B**) Average coulombic capture efficiency (CCE) associated with acetate and methane production. Results are reported for reactors operated at −0.7 V vs. SHE and −0.9 V vs. SHE by using bioaugmented and non-bioaugmented mixed-culture inocula. Values are reported as mean ± standard deviation.

In contrast, operation at −0.9 V vs. SHE produced substantially different behaviours. In the absence of bioaugmentation for both case, methane accumulated throughout reactor operation reaching significantly higher concentrations (around 2 mmol/L) than those observed under the −0.7 V potential (around 0.25 mmol/L). Acetate was also produced, but methane represented a major fraction of the recovered carbon. Conversely, the bioaugmented reactor operated at −0.9 V vs. SHE showed a progressive decrease in methane concentration after approximately day 15, stabilizing at 0.4 mmol/L with a CCE of 5%, while acetate concentrations remained relatively stable during the subsequent semi-continuous phase, obtaining around 2.15 mmol/L and a CCE of 43%. The concentration profiles of secondary carboxylic acids further confirmed the influence of both cathodic potential and bioaugmentation on product distribution. Overall, the temporal concentration profiles indicate that the combination of cathodic potential and bioaugmentation strongly influenced the stability and selectivity of CO_2_ conversion during semi-continuous bioelectrochemical operation, with −0.7 V vs. SHE favouring stable acetogenic conditions and bioaugmentation mitigating methane accumulation under the more reducing −0.9 V condition, as it is also shown in Figure 3, where the average values in terms of production rates (Figure 3A), and CCE average (Figure 3B) obtained are reported.

At −0.7 V vs. SHE, acetogenesis clearly prevailed over methanogenesis regardless bioaugmentation. Acetate production rates were comparable in the non-bioaugmented and bioaugmented reactors, reaching 0.0385 ± 0.01 and 0.0343 ± 0.01 mmol d^−1^, respectively, corresponding to CCE values of 68% and 64%. Conversely, methane accounted for only a minor fraction of the recovered electrons, with a CCE equal to 2.3% and 8.8%, considering the bioaugmented inoculum or not, respectively. While most of the reducing equivalents were recovered in acetate and when secondary carboxylic acids were also considered, total acid production reached its highest value under the −0.7 V vs. SHE conditions, with a CCE of 70.6% and 79.5%, by working with bioaugmented or non-bioaugmented reactors, respectively.

In contrast, operation at −0.9 V vs. SHE shifted product selectivity towards methanogenesis in the absence of bioaugmentation. Indeed, the non-bioaugmented reactor exhibited the highest methane production rate of the entire study (0.105 ± 0.012 mmol d^−1^), corresponding to a CCE of 58%, whereas acetate production decreased to 0.0728 ± 0.025 mmol d^−1^ (CCE = 36%). Bioaugmentation substantially modified this behavior; in presence of *A. woodii*, methane production decreased by more than one order of magnitude (0.007 ± 0.004 mmol d^−1^; CCE = 5%), while acetate production remained relatively high (0.061 ± 0.025 mmol d^−1^; CCE = 43%). Also, although secondary acids remained minor products for all configurations tested, their cumulative production was more pronounced in the bioaugmented reactor, with a total acids CCE equal to 50.5% against the 41% reached in the non-bioaugmented H-cell operated at −0.9 V vs. SHE, confirming that bioaugmentation effectively redirected electron flux towards acetogenic metabolism under highly reducing conditions.

Table 2 and Table 3 provide the main results obtained for all the tests carried out, in terms of current generated and CCE and production rate, respectively.

**Figure 4:**
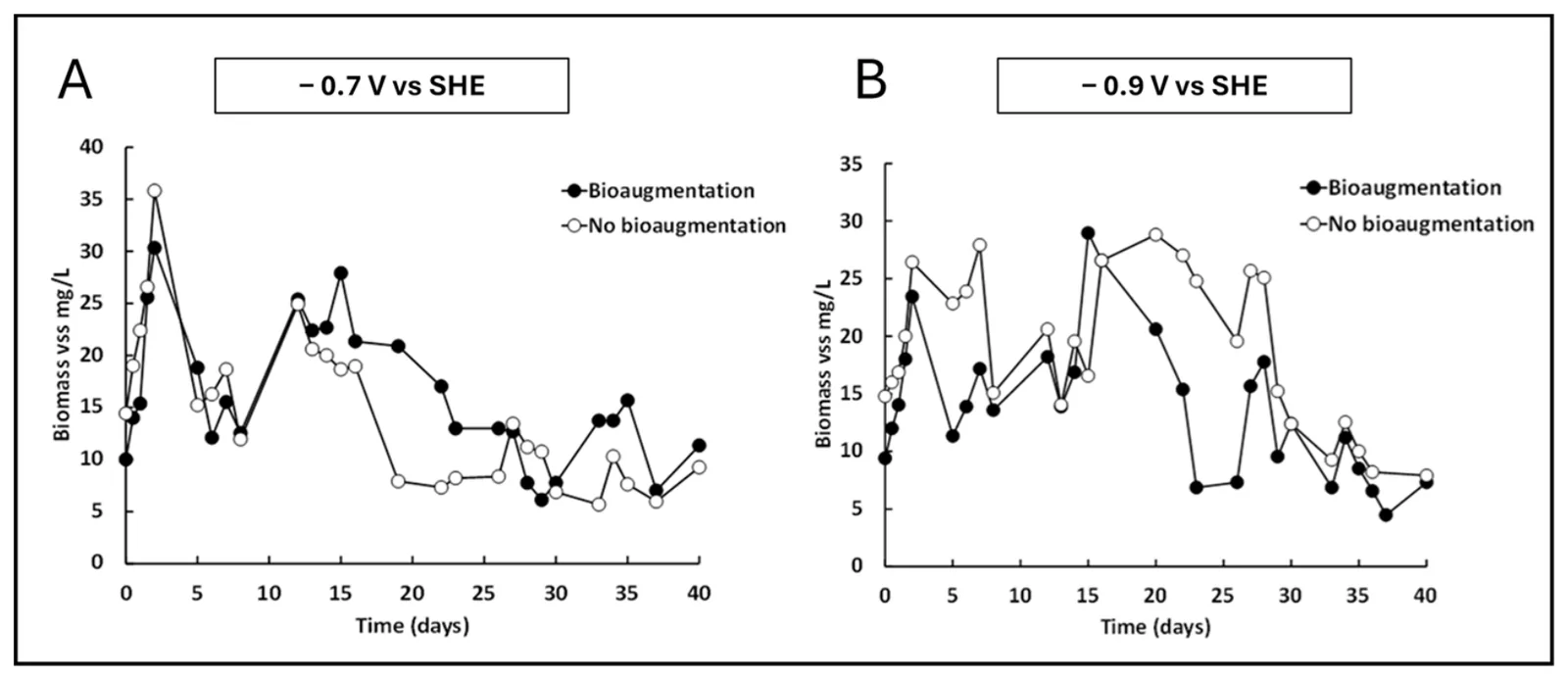
Temporal evolution of biomass concentration during semi-continuous operation of the H-cell bioelectrochemical reactors. (**A**) Reactors operated at −0.7 V vs. SHE under bioaugmented and non-bioaugmented conditions. (**B**) Reactors operated at −0.9 V vs. SHE under bioaugmented and non-bioaugmented conditions.

The temporal evolution of biomass concentration further highlighted the different reactor behaviors under the operating conditions investigated (Figure 4). In all reactors, an initial increase in suspended biomass concentration was observed during the first 10–15 days of operation, indicating active microbial growth after reactor start-up.

At −0.7 V vs. SHE (Figure 4A), both the bioaugmented and non-bioaugmented reactors exhibited a progressive decrease in suspended biomass concentration after approximately day 15–20, with values declining from about 20–30 mg VSS L^−1^ to below 10 mg VSS L^−1^ by the end of the experiment. Despite this decrease in bulk biomass, acetate production remained relatively stable and methane production remained minimal under this operating condition.

In contrast, reactors operated at −0.9 V vs. SHE (Figure 4B) maintained higher suspended biomass concentrations for a longer period. The non-bioaugmented reactor showed the most persistent biomass accumulation, with concentrations remaining above 20 mg VSS L^−1^ for a substantial portion of the experimental period, which was consistent with the higher methane production observed under this condition. The bioaugmented reactor also exhibited active biomass growth during the first half of the experiment, followed by a gradual decrease in suspended biomass concentration during the later stages of operation.

Overall, the biomass profiles indicate that the applied cathodic potential influenced not only product selectivity but also the retention and distribution of active biomass within the bioelectrochemical system. The stronger reduction in suspended biomass observed at −0.7 V vs. SHE, together with the sustained acetate production, suggests that a larger fraction of the metabolically active community may have become associated with the electrode surface rather than remaining suspended in the bulk liquid.

**Figure 5:**
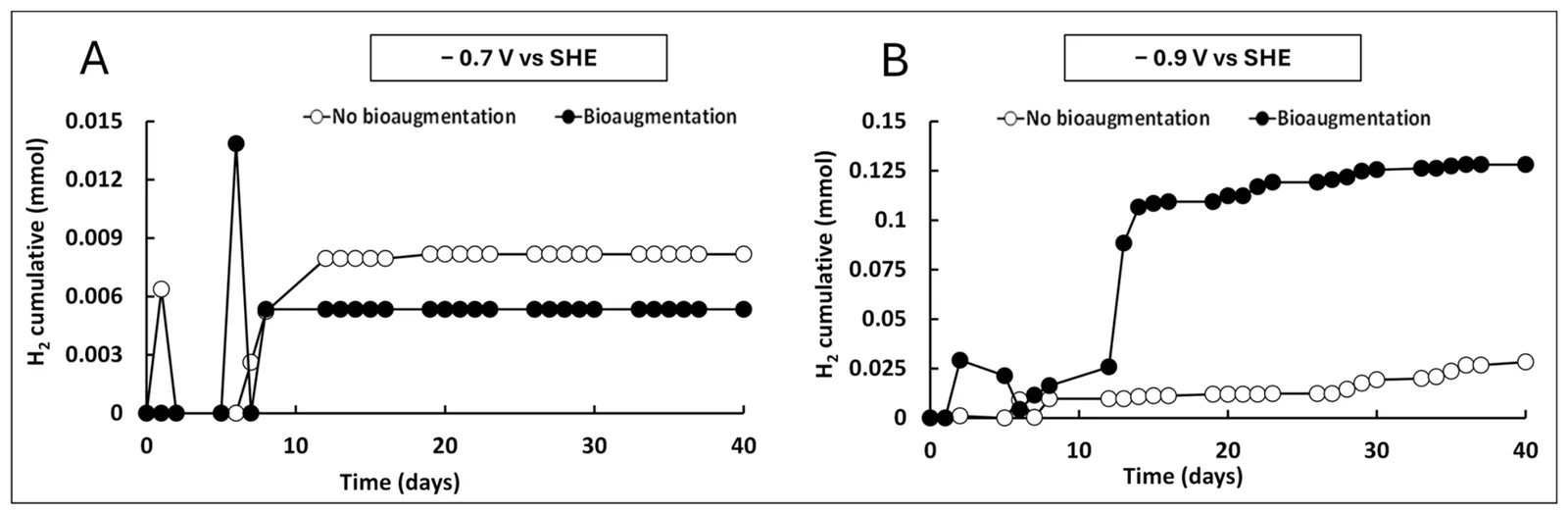
Cumulative hydrogen production, calculated from daily headspace gas composition analyses, during semi-continuous operation of the H-cell bioelectrochemical reactors. (**A**) Reactors operated at −0.7 V vs. SHE under bioaugmented and non-bioaugmented conditions. (**B**) Reactors operated at −0.9 V vs. SHE under bioaugmented and non-bioaugmented conditions.

Figure 5 show the cumulative hydrogen detected in the headspace of the H-cell reactors during semi-continuous operation under the two cathodic potentials investigated. Under the −0.7 V vs SHE conditions (Figure 5A), hydrogen accumulation remained extremely low in both the bioaugmented and non-bioaugmented reactors, reaching stable plateau values below 0.01 mmol after the first week of operation. This behaviour suggests that electrochemically generated hydrogen was rapidly consumed by the microbial community and did not significantly accumulate in the reactor headspace.

In contrast, operation at −0.9 V vs. SHE (Figure 5B) resulted in a substantially higher hydrogen accumulation, particularly in the bioaugmented reactor, where the cumulative amount increased sharply after approximately 12 days and reached about 0.13 mmol by the end of the experiment. The non-bioaugmented reactor also showed a gradual increase in hydrogen accumulation, although the final amount remained markedly lower than in the bioaugmented condition.

These results indicate that the applied cathodic potential strongly influenced hydrogen availability in the bioelectrochemical system [40]. The limited hydrogen accumulation observed at −0.7 V vs. SHE is consistent with the stable acetate and other products observed under this condition, whereas the higher hydrogen accumulation at −0.9 V vs. SHE suggests that the more reducing potential promoted hydrogen evolution beyond the consumption capacity of the microbial community. This pronounced hydrogen accumulation observed in the bioaugmented reactor at −0.9 V vs. SHE, despite the lower methane production measured under this condition, further supports the hypothesis that bioaugmentation altered the competition for reducing equivalents within the cathodic biofilm, favoring acetogenic activity over hydrogenotrophic methanogenesis.

Overall, these results demonstrate that both cathodic potential and bioaugmentation significantly influenced the selectivity of CO_2_ conversion, with −0.7 V vs. SHE better promoting acetogenesis, while bioaugmentation with *A. woodii* effectively mitigated methanogenesis under the more reducing −0.9 V operating condition.

### 3.2. Electrochemical Characterization of the Biocathodes

**Figure 6:**
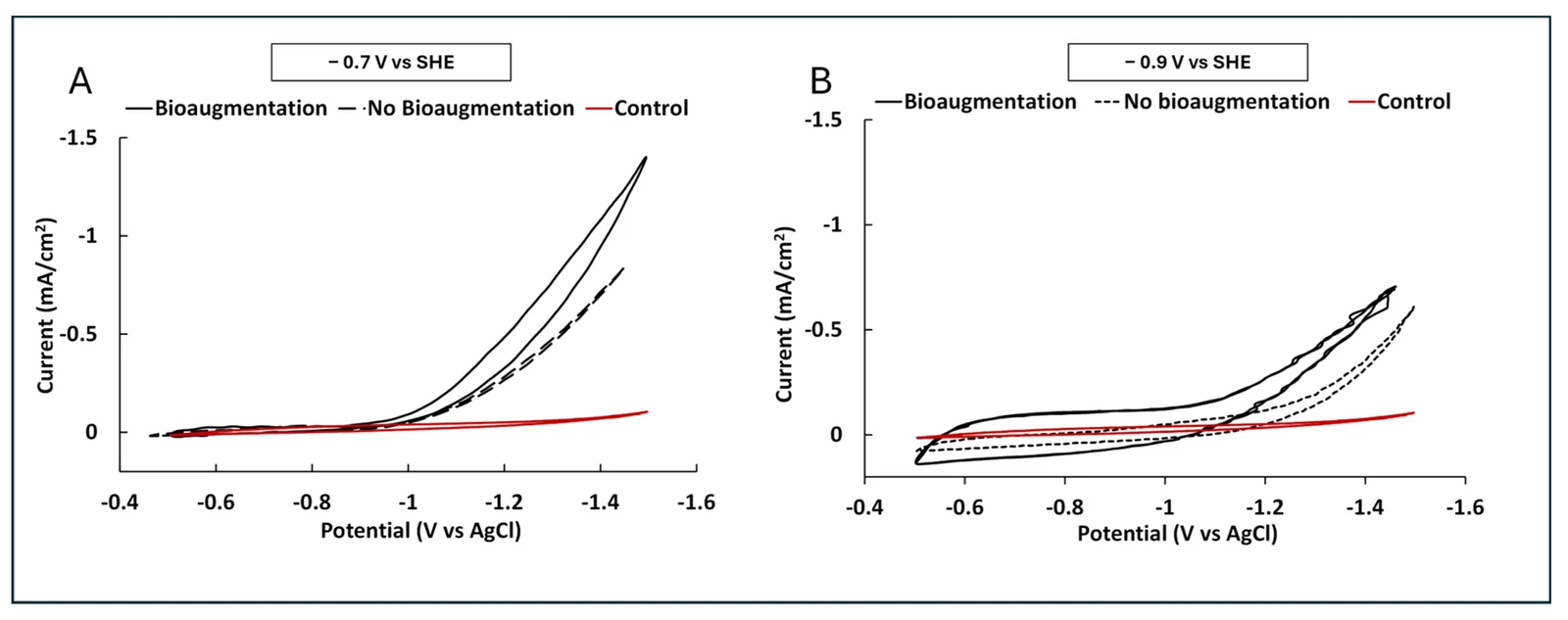
Cyclic voltammetry profiles of the developed biocathodes at the end of reactor operation. (**A**) Voltammetric responses obtained from reactors operated at −0.7 V vs. SHE under bioaugmented and non-bioaugmented conditions and (**B**) voltammetric responses obtained from reactors operated at −0.9 V vs. SHE under bioaugmented and non-bioaugmented conditions, together with the respective abiotic control electrode.

Cyclic voltammetry was performed at the end of the experimental tests to evaluate the electrochemical behavior of the developed biocathodes and to assess the effect of bioaugmentation on current evolution activity. Representative cyclic voltammograms obtained under abiotic conditions and after biofilm development are shown in Figure 6.

The abiotic electrode exhibited the characteristic onset of current evolution at approximately −1.3 V vs. SHE. In contrast, all biotic electrodes displayed enhanced cathodic currents, confirming the development of electroactive biofilms capable of catalyzing hydrogen evolution.

At the operating potential of −0.7 V vs. SHE (Figure 6A), the bioaugmented biocathode exhibited higher cathodic currents than the non-bioaugmented reactor over the investigated potential range. Moreover, the larger capacitive response observed for the bioaugmented electrode suggests the development of a more extensive biofilm, as previously correlated with increased biofilm coverage on electrode surfaces. Similar improvements in current evolution catalysis have been reported for Acetobacterium-dominated biocathodes operating under comparable cathodic potentials [41].

Different electrochemical behavior was observed at −0.9 V vs. SHE (Figure 6B). Under these conditions, both inocula generated comparable catalytic responses, although the bioaugmented electrode still exhibited a higher capacitive current, suggesting enhanced biofilm development without a corresponding increase in catalytic activity.

However, comparison between the two operating potentials revealed that the onset of bioelectrocatalytic current occurred at less negative potentials in the reactors operated at −0.7 V vs. SHE, indicating a lower energetic requirement for hydrogen evolution. This behavior is consistent with the superior acetogenic performance observed under this operating condition.

## 4. Discussion

### 4.1. Operating Conditions Promoted Selective Acetogenesis Without Chemical Methanogenic Inhibitors

One of the most relevant findings of this study is that selective acetogenesis was successfully promoted in mixed microbial cultures without the addition of chemical methanogenic inhibitors. In particular, the reactors operated at −0.7 V vs. SHE showed sustained acetate production accompanied by negligible methane formation, demonstrating that process selectivity and the development of conductive and efficient biofilm can be effectively controlled through operational parameters, as the applied potential, rather than by relying on compounds such as 2-bromoethanesulfonate [42]. The suppression of methanogenesis observed under the −0.7 V conditions is particularly significant because methanogens generally outcompete acetogens during hydrogen-mediated CO_2_ reduction owing to the greater thermodynamic favourability of methane formation [10,43]. Previous studies on MES have frequently employed BES as a selective inhibitor to prevent methane production and favour acetate accumulation in mixed cultures [12,44]. Although effective, this strategy increases operating costs and may affect the stability of the microbial community, thereby limiting its practical applicability for large-scale bioelectrochemical processes.

In the present work, methanogenesis was controlled exclusively through the combination of inoculum pretreatment, hydraulic dilution rate and cathodic potential. In this view, thermal and acid pretreatments have previously been reported to temporarily suppress methanogenic populations while enriching spore-forming acetogenic microorganisms [31]. However, most available studies have demonstrated that methanogenesis tends to recover after a relatively short period under batch operation, often becoming dominant again once methanogens regain activity [45,46]. While the sustained low methane concentrations observed during semi-continuous operation in this study suggest that the applied operating conditions maintained a selective pressure favourable to acetogenic metabolism over the entire experimental period. Hence, the role of the dilution strategy appears particularly important. The reactors were operated at a D rate corresponding to 50% of the experimentally determined μ_max_, thereby maintaining active biomass growth while continuously removing suspended microorganisms from the bulk liquid.

The applied cathodic potential further contributed to shaping the competition between acetogenic and methanogenic pathways. Although similar initial growth kinetics were observed at both −0.7 and −0.9 V vs. SHE, markedly different product distributions were obtained during semi-continuous operation. The reactors operated at −0.7 V vs. SHE maintained stable acetate production with minimal methane accumulation, whereas the more reducing −0.9 V condition favoured methane formation in the absence of bioaugmentation. This behaviour is consistent with previous studies showing that *Acetobacterium*-dominated biocathodes can enhance biocathodic activity and facilitate electron transfer between the electrode and the microbial community [47]. Moreover, increasingly negative cathodic potentials favour the electrochemical evolution of H_2_, thereby increasing the availability of H_2_ as a reducing equivalent for hydrogenotrophic microorganisms, including methanogens [48,49].

Under the less reducing −0.7 V condition, hydrogen availability may have remained sufficiently low to limit methanogenic activity while still supporting acetogenic CO_2_ reduction. In contrast, the substantial methane production observed at −0.9 V vs. SHE suggests that the higher reducing power supplied to the cathode promoted methanogenic electron utilization when no additional selective pressure was present. Interestingly, despite the lower thermodynamic driving force associated with −0.7 V vs. SHE, this condition resulted in the highest acetogenic bacteria selectivity and the lowest methane production. These results indicate that maximizing the reducing power supplied to the cathode does not necessarily improve acetogenic performance in mixed-culture BES, and that moderate cathodic potential may provide a more favourable balance between electron availability and process selectivity.

### 4.2. Bioaugmentation with Acetobacterium woodii Enhanced Process Stability and Biocathode Development

Although bioaugmentation did not substantially increase acetate production under all the investigated conditions, it significantly influenced process stability under the most reducing cathodic potential (−0.9 V vs. SHE). The comparison between the bioaugmented and non-bioaugmented reactors operated at −0.9 V showed a marked reduction in methane production following the addition of *Acetobacterium woodii*, while acetate production remained relatively stable. This behaviour suggests that bioaugmentation modified the competition between acetogenic and methanogenic populations, favouring the maintenance of acetogenic activity under conditions that otherwise promoted methane formation.

The effect of bioaugmentation was less evident at −0.7 V vs. SHE, where methane production was already strongly suppressed by the operating conditions. In contrast, the pronounced difference observed at −0.9 V indicates that the additional selective pressure introduced by *A. woodii* became particularly relevant when hydrogen availability was presumably higher and methanogens could more easily compete for reducing equivalents. Similar observations have been reported in MES systems where *Acetobacterium* species became dominant members of cathodic biofilms and contributed to enhanced acetogenic performance [20,50]. The temporal concentration profiles further support the stabilizing effect of bioaugmentation. In the bioaugmented reactor operated at −0.9 V, methane concentrations progressively decreased after approximately day 15, whereas acetate concentrations remained comparatively stable during the subsequent semi-continuous phase. Although the microbial community attached to the cathode was not directly characterized in this study, these observations are consistent with the establishment of a biofilm more favourable to acetogenic metabolism than to methanogenic growth.

Additional evidence was provided by the cyclic voltammetry analyses performed at the end of reactor operation. Under the −0.7 V condition, the bioaugmented biocathode exhibited higher cathodic currents and a larger capacitive response than the non-bioaugmented reactor. Increased capacitance is commonly associated with greater biofilm coverage and enhanced electroactive biomass development on electrode surfaces [51,52]. The earlier onset of cathodic current observed in the bioaugmented electrode also suggests improved catalytic activity for hydrogen evolution, which could increase the local availability of reducing equivalents for acetogenic microorganisms.

At −0.9 V vs. SHE, both biocathodes showed comparable catalytic current responses, indicating that the strongly reducing potential itself promoted substantial electrochemical activity. Nevertheless, the bioaugmented electrode still displayed a higher capacitive current, suggesting a greater propensity for biofilm formation even when differences in catalytic activity were less pronounced. However, the structure and spatial organization of the biofilm are important factors, to be considered in future work, since can influence microbial interactions, electron transfer processes, and, consequently, overall system functionality [53].

Taken together, these results indicate that *A. woodii* bioaugmentation primarily contributed to the development of a more stable and electroactive biocathode rather than simply increasing acetate production rates. The combined evidence from methane suppression, temporal concentration profiles and cyclic voltammetry suggests that bioaugmentation enhanced the establishment of cathode-associated acetogenic activity, thereby improving process robustness under highly reducing bioelectrochemical conditions.

### 4.3. Implications for Sustainable CO_2_ Valorisation in Mixed-Culture BES

The results obtained in this study have important implications for the development of sustainable bioelectrochemical platforms for CO_2_ valorisation and resource recovery. Indeed, the present work demonstrates that selective acetogenesis can be promoted in mixed microbial cultures through the combined optimization of inoculum pretreatment, D rate, and cathodic potential, without working in sterile conditions, with pure strains or adding chemical inhibitors. The use of mixed cultures represents a significant advantage for practical applications because it avoids the need for strict sterilization procedures and generally provides greater operational robustness when treating complex waste-derived streams [54].

Another relevant aspect is that the proposed strategy relies on operational parameters that can be readily adjusted in continuous systems, such as D rate, HRT and electrode potential. This approach is potentially more compatible with process scale-up than strategies based on continuous inhibitor addition, particularly in the context of wastewater treatment and industrial CO_2_-containing gas streams, where minimizing chemical inputs is essential for economic and environmental sustainability [55]. Furthermore, the production of acetate and secondary carboxylic acids from CO_2_ represents an attractive route for carbon capture and utilization within a circular bioeconomy framework. Acetate is a versatile platform chemical that can be directly recovered or further converted into higher-value products, including medium-chain carboxylic acids, biopolymers, and other bio-based chemicals. Therefore, steering MMC bioelectrochemical systems toward acetogenic pathways may enable the simultaneous mitigation of CO_2_ emissions and generation of valuable products.

Overall, this study provides evidence that process-based control strategies can effectively direct CO_2_ reduction toward acetogenesis in semi-continuous mixed-culture BES without the use of chemical methanogenic inhibitors. These findings contribute to the development of more sustainable and economically viable bioelectrochemical technologies for carbon capture, CO_2_ valorisation, and resource recovery from waste-derived streams.

## 5. Conclusions

This study investigated the selective conversion of CO_2_ in semi-continuous MMC bioelectrochemical systems operated without chemical methanogenic inhibitors. The combined strategy based on inoculum pretreatment, controlled dilution rate, and cathodic potential successfully promoted acetogenic activity while limiting methane formation. Among the investigated operating conditions, the cathodic potential of −0.7 V vs. SHE resulted in the highest acetate selectivity and the lowest methane production, demonstrating that moderate reducing potentials can favour acetogenesis over methanogenesis in mixed-culture BES. In contrast, operation at −0.9 V vs. SHE promoted methane formation in the absence of bioaugmentation, highlighting the critical role of cathodic potential in determining process selectivity. Bioaugmentation with *Acetobacterium woodii* significantly improved process stability under the highly reducing −0.9 V condition by strongly reducing methane accumulation while maintaining acetate production. Cyclic voltammetry analyses further suggested that bioaugmentation enhanced biocathode development and electrochemical activity, supporting the establishment of a more stable acetogenic biofilm. Overall, the obtained results demonstrate that selective acetogenesis can be achieved in mixed microbial cultures through process-based operational control without the addition of BESA. Nevertheless, the proposed operational strategy provides a scalable and inhibitor-free framework for steering mixed-culture bioelectrochemical systems toward the production of value-added carboxylic acids from CO_2_-containing waste streams.

## Figures and Tables

**Table 1:** Electrochemical performances of the different H-cells operated with −0.7 and −0.9 V vs. SHE, under bioaugmented and non-bioaugmented conditions.

V vs. SHE	Inoculum	Current (mA)	Current Density (mA/cm^2^)	Cell Voltage (V)
−0.7	Bioaug.	−0.52 ± 0.08	−0.04 ± 0.01	−1.58 ± 0.01
	No bioaug.	−0.48 ± 0.08	−0.04 ± 0.01	−1.71 ± 0.57
−0.9	Bioaug.	−1.85 ± 0.08	−0.12 ± 0.01	−3.07 ± 0.68
	No bioaug.	−1.62 ± 0.04	−0.16 ± 0.01	−3.43 ± 0.52

**Table 2:** Average values in terms of cathodic coulombic efficiencies and produced current, obtained for H-cells operated with −0.7 and −0.9 V vs. SHE, under bioaugmented and non-bioaugmented conditions. Values are reported as mean ± standard deviation.

		CCE (%)						
V vs. SHE-	Inoculum	Acetic	Propionic	IsoBut/Butyric	Valeric	CH_4_	H_2_	Global
−0.7	Bioaugmentation	64 ± 2	2.7 ± 0.3	1.8 ± 0.4	2.1± 0.3	2.3 ± 0.3	2.3 ± 0.3	19 ± 1
	No Bioaugmentation	68 ± 1	4.5 ± 0.2	3.3 ± 0.3	3.7 ± 0.5	8.8 ± 0.2	2.3 ± 0.3	20 ± 1
−0.9	Bioaugmentation	43 ± 1	2.7 ± 0.3	2.4± 0.2	2.3 ± 0.2	5.2 ± 0.1	2.3 ± 0.3	2.3 ± 0.3
	No Bioaugmentation	36 ± 1	1.9 ± 0.1	1.6 ± 0.1	1.4 ± 0.2	58 ± 0.3	2.3 ± 0.3	6.2 ± 0.5

**Table 3:** Average production rates observed for the different species observed, obtained for H-cells operated with −0.7 and −0.9 V vs. SHE, under bioaugmented and non-bioaugmented conditions.

		Production Rate (mmol d^−1^)					
V vs. SHE	Inoculum	Acetic	Propionic	Iso/Butyric	Valeric	CH_4_	H_2_
−0.7	Bioaugmentation	0.0343 ± 0.0002	0.0010 ± 0.0002	0.0005 ± 0.0001	0.0004 ± 0.0002	0.0012 ± 0.0003	0.0001 ± 0.0001
	No Bioaugmentation	0.0385 ± 0.0001	0.0014 ± 0.0002	0.0008 ± 0.0003	0.0007 ± 0.0001	0.0039 ± 0.0008	0.0002 ± 0.0001
−0.9	Bioaugmentation	0.0613 ± 0.0001	0.0028 ± 0.0003	0.0017 ± 0.0002	0.0013 ± 0.0002	0.0073 ± 0.0006	0.0032 ± 0.0003
	No Bioaugmentation	0.0728 ± 0.0001	0.0026 ± 0.0001	0.0016 ± 0.0001	0.0012 ± 0.0002	0.1050 ± 0.0015	0.007 ± 0.0002

## Data Availability

The original contributions presented in this study are included in the article. Further questions can be directed at the corresponding author.
